# Functional Equivalence of Insulin and IGF-1 in the In Vitro Culture of Chicken Primordial Germ Cells

**DOI:** 10.3390/genes16050481

**Published:** 2025-04-24

**Authors:** Xin Liu, Jun Wu, Yixiu Peng, Guangzheng Liu, Kai Jin, Yingjie Niu, Jiuzhou Song, Wei Han, Guohong Chen, Bichun Li, Qisheng Zuo

**Affiliations:** 1 Key Laboratory of Animal Genetics, Breeding and Molecular Design of Jiangsu Province, College of Animal Science and Technology, Yangzhou University, Yangzhou 225009, China; 2 Joint International Research Laboratory of Agriculture and Agri-Product Safety of Ministry of Education of China, Yangzhou University, Yangzhou 225009, China; 3 Animal & Avian Sciences, University of Maryland, College Park, MD 20742, USA; 4 Poultry Institute, Chinese Academy of Agricultural Sciences Poultry Institute of Jiangsu, Yangzhou 225003, China; 5 College of Biotechnology, Jiangsu University of Science and Technology, Zhenjiang 212100, China

**Keywords:** chicken PGCs, IGF-1, migration, establishment efficiency

## Abstract

Background: Chicken Primordial Germ Cells (PGCs) are one of the few germ cells that can be cultured for a long time in vitro, but challenges remain such as low culture efficiency and unclear roles of nutrient factors and signaling pathways. Method: In this study, protein kinase B (AKT) pathway activator insulin-like growth factor 1 (IGF-1) was screened for its ability to promote cell proliferation by transcriptome results using various inhibitors of pathway activation. The effects of IGF-1 on PGCs were evaluated through EdU assays, qRT-PCR, flow cytometry, and migration experiments. Results: This study systematically examined the effects of insulin and IGF-1 on the proliferation, cell cycle, ferroptosis, migration capacity, and establishment efficiency of PGCs. The findings demonstrated that IGF-1 exhibited comparable effects to insulin and could effectively replace insulin in PGC culture systems. Conclusions: The research results are expected to provide a solid theoretical basis for optimizing the chicken PGC cultivation system and promoting its practical application.

## 1. Introduction

Avians are considered optimal animal models for developmental biology research due to the feasibility of in vitro culture and their distinct evolutionary position. These characteristics offer significant advantages for investigating embryonic development, cellular differentiation, and gene regulation. Furthermore, avian models are indispensable in reproductive biology, genetic breeding, and transgenic technology research [1,2,3]. Owing to the numerous advantages of avian species, the practical application of avian primordial germ cells (PGCs) has garnered widespread attention. After years of research and optimization, chicken PGCs have emerged as one of the few reproductive cell types capable of long-term in vitro culture. However, challenges remain, including low establishment efficiency and a limited understanding of the roles of various nutritional factors within the culture system [4]. Whyte et al. established a culture system that enables the self-renewal of PGCs using fibroblast growth factor 2 (FGF2), Activin A, and insulin, eliminating the need for feeder cells and serum [5].

The defined culture medium conditions provide valuable insights into the key molecular pathways essential for PGC self-renewal. Insulin is a protein hormone consisting of 51 amino acid residues, secreted by pancreatic β cells in response to stimulation within the human body [6,7]. Insulin plays a pivotal role in a multitude of physiological processes and interacts with other hormones and growth factors through a complex signaling network. Together, these factors contribute to maintaining the stability of the internal environment. The transmission of insulin signaling is primarily facilitated by two pathways: mitogen-activated protein kinase (MAPK) and phosphatidylinositol 3-kinase (PI3K) [8]. The MAPK signaling pathway is a critical component of diverse cellular processes, including cell proliferation, differentiation, apoptosis, and basic metabolism. This pathway plays a pivotal role in maintaining cellular homeostasis and contributes to a variety of physiological functions [9]. PI3K/AKT signaling has been demonstrated to have the capacity to impede cell apoptosis that has been triggered by a variety of stimuli associated with apoptosis, while concomitantly promoting cell survival during the process of apoptosis in a range of cell types [10,11,12]. IGF-1 and insulin exhibit structural and functional similarity [13]. However, the optimal insulin concentration required for PGC cultivation has not yet been determined. Notably, previous studies have shown that BMP4 can replace Activin A under non-cloning conditions, but its effect varies during cloning amplification [5]. So far, the full potential of IGF-1 as a complete replacement for insulin in PGC culture systems has not been systematically evaluated.

Based on the above issues, the study initially conducted a comprehensive analysis of the synergistic effects between IGF1 and other small molecule compounds using transcriptome data analysis in the early stage. Next, the study proceeded to conduct in-depth research on the effects of insulin and IGF1 on chicken PGCs proliferation, cycle, iron death, migration, and establishment efficiency. The findings from this study offer a more robust theoretical foundation for the development of a more comprehensive chicken PGC cultivation system and its subsequent applications.

## 2. Materials and Methods

### 2.1. PGC Culture Conditions

All experimental procedures in this study were approved by the Experimental Animal Ethics Committee of Yangzhou University [approval number: SYXK (Su) 2021-0027]. The isolation and cultivation of PGCs were conducted with reference to previous studies [14,15]. PGCs were obtained from Rugao Yellow Chicken embryos at Hamburger and Hamilton (HH) stages 27–28. Following dissection, the gonads were rinsed with 0.1% BSA, mechanically dissociated, and enzymatically digested with Accutase for 7 min to facilitate the release of primordial germ cells. The resulting cell suspension was centrifuged, and the pellet was resuspended in 200 μL of FAcs culture medium (Supplementary Table S1). Finally, the PGCs were plated in a 48-well dish and incubated at 37 °C under 5% CO_2_ conditions.

### 2.2. Preparation and Treatment of Small Molecule Compounds

PGCs in the logarithmic growth phase were inoculated into 24-well plates (1 × 10^5^). Negative controls (insulin-free) were treated with B-27™ Plus supplement (50×) (Thermo Fisher Scientific, Waltham, MA, USA), while positive controls (insulin) were treated with B-27™ minus insulin (Thermo Fisher Scientific, USA). The small-molecule compound IGF-1 (HY-P7018; MedChemExpress, Monmouth, NJ, USA) was dissolved in phosphate-buffered saline (PBS) at a concentration of 100 ng/mL. The remaining small-molecule compounds were dissolved in Dimethyl Sulfoxide (DMSO) solvent with the following product codes and concentrations: LY294002 (HY-10108; 40 μM); RAS inhibitor Abd-7 (HY-122862; 20 μM); ML-099 (HY-124306; 100 nM); MHY1485 (HY-B0795; 20 μM); Rapamycin (HY-10219; 100 nM); Pyrintegrin (HY-13306; 10 µM); and BIRT 377 (HY-110117; 100 nM).

### 2.3. Cell Counting Kit-8 (CCK-8) Assay

In this assay, 10 μL of CCK-8 reagent was added to 100 μL of the culture medium, with three parallel wells established for the experimental condition. The samples were then subjected to an incubation period of 2 h at 37 °C within a 5% CO_2_ incubator. Following this, the samples were measured for their absorbency at a wavelength of 450 nm using a SPARK spectrophotometer (Otsuka, Osaka, Japan). The data statistical analysis refers to the Cell Counting Kit (CCK-8) (Dojindo, Mashiki, Japan).

### 2.4. EdU Proliferation Assay

EdU was diluted in PGC complete medium (1000:1) to prepare 50 μM EdU medium, and 100 μL was added to each well for incubation for 2 h. After discarding the medium, the cells were washed with PBS, centrifuged (1400 rpm, 6 min), and resuspended in 20 μL of the remaining supernatant before being placed on glass slides. Cells were fixed with 4% paraformaldehyde for 30 min, washed, and treated with 2 mg/mL glycine for 5 min. Permeabilization was performed with 0.5% TritonX-100 in PBS for 10 min. After washing, 1× Apollo staining solution was added for 30 min in the dark, followed by multiple washes. Hoechst 33,342 staining was then applied for 30 min in the dark. After final washing and permeabilization, the cells were mounted with neutral gum for analysis.

### 2.5. RNA Extraction and Real-Time Quantitative PCR (RT–qPCR)

PGCs were collected, centrifuged (1400 rpm, 6 min), and lysed with TRIZOL. After phase separation with chloroform (12,000× *g*, 4 °C, 15 min), the aqueous phase containing RNA was extracted and precipitated with isopropanol. The RNA pellet was washed with 75% ethanol, air dried, and dissolved in enzyme-free water. RNA concentration and purity (OD260/OD280) were measured. Reverse transcription was performed using 5×FastKing-RT SuperMix with total RNA (50 ng–2 μg) in a 20 μL reaction (42 °C for 15 min, 95 °C for 3 min). qRT-PCR was performed using a 10 μL system containing SYBR Green qPCR Mix, primers, and cDNA. Reaction conditions were 95 °C for 3 min, followed by 40 cycles of 95 °C for 5 s and 60 °C for 30 s. Three biological and technical replicates were performed for each PGC sample. Gene expression was quantified using the 2^−ΔΔCT^ method after normalization to the mRNA expression of the housekeeping gene β-actin. The primers used are listed in the Supplementary Material (Table S1).

### 2.6. Detection of Apoptosis by the FITC/PI Double Staining Method

After centrifugation at 1000 rpm for 5 min, the cells were collected, washed twice with precooled PBS, and resuspended to obtain 5 × 10^5^ cells. Then, 100 μL of 1× binding buffer was added, followed by 5 μL of Annexin V-FITC and 10 μL of PI staining solution. The mixture was incubated in the dark at room temperature for 15 min. Afterward, 400 μL of 1× binding buffer was added, and the sample was analyzed by flow cytometry. Detailed procedures followed the instructions of the Annexin V-FITC/PI Apoptosis Detection Kit (Yeasen Biotechnology, Shanghai, China). The assay distinguished live, dead, early apoptotic, and apoptotic cells.

### 2.7. Reactive Oxygen Species Assay

Cells were collected by centrifugation and incubated with 10 μM DCFH-DA at 37 °C for 2 h, with mixing every 10 min. After incubation, they were washed with a serum-free culture solution to remove excess DCFH-DA that had not entered the cells. Reactive oxygen species (ROS) levels were then analyzed by flow cytometry. For detailed procedures, refer to the Reactive Oxygen Species (ROS) Assay Kit (Yeasen Biotechnology, China).

### 2.8. Cell Cycle Analysis

Cell cycle analysis was performed through flow cytometry. Cells were first fixed by gently mixing the pellet with 1 mL of pre-cooled 70% ethanol, followed by incubation at 4 °C overnight. After centrifugation (1000× *g*, 5 min), the pellet was washed with pre-cooled PBS and centrifuged again under the same conditions. A propidium iodide (PI)-staining solution was prepared by mixing 10 μL PI stock solution and 10 μL RNase A with 0.5 mL staining buffer. Each cell sample was resuspended in 0.5 mL of the prepared PI staining solution, gently mixed, and incubated for 30 min at 37 °C in the dark. Finally, samples were analyzed with flow cytometry at an excitation wavelength of 488 nm, and DNA content and light scatter were assessed using FlowJo software (FlowJo_v10.8.1; BD, Ashland, Jackson, OR, USA).

### 2.9. Assay for GSH/GSSG/MDA/Fe^2+^

For Fe^2+^ detection, 1 × 10^6^ PGCs were lysed on ice for 10 min, followed by centrifugation (15,000× *g*, 10 min) to collect the supernatant. Standards and samples (80 μL each) were added to a microplate, with reagent 2 added to control wells and reagent 3 to assay and standard wells. After incubation at 37 °C for 10 min, absorbance was measured at 593 nm.

For GSH and GSSG assays, the content of reduced glutathione (GSH) and oxidized glutathione disulfide (GSSG) was determined using a GSH and GSSG assay kit (Beyotime, Haimen, China), following the manufacturer’s instructions. The GSH content of test samples was calculated as follows: total glutathione − (GSSG × 2). Cells were centrifuged (1400 rpm, 6 min) and plated in a 96-well plate. After adding samples or standards, 150 μL of the GSH assay working solution was mixed in, followed by incubation for 5 min. Then, 50 μL of 0.5 mg/mL NADPH solution was added and mixed, and absorbance was recorded at 405 nm.

For the MDA assay, malondialdehyde (MDA) levels were measured using a lipid peroxidation MDA assay kit (KeyGEN BioTECH, Nanjing, China), following the manufacturer’s protocol. After centrifugation (12,000× *g*, 4 °C, 10 min), the supernatant was collected and analyzed using a microplate reader at 532 nm. Additionally, 1 × 10^6^ cells were lysed on ice for 20 min with intermittent shaking, centrifuged (12,000× *g*, 5 min), and mixed with double-distilled water. The sample was boiled for 50 min, rapidly cooled in ice water, and centrifuged (3000 rpm, 4 °C, 15 min), and the supernatant was tested.

### 2.10. Detection of PGC Migration

This experimental method is based on the work of Niu et al. [14]. When the chicken embryo has developed to 2.5 days (HH 14–16), it is disinfected with alcohol and a small hole of about 1 cm in diameter is drilled at the blunt end of the egg. Approximately 5000 green fluorescent protein-labeled primordial germ cells (GFP-PGCs) are injected into the dorsal aortic vessels of chicken embryos under a stereomicroscope (GFP-PGC was constructed by our laboratory, with specific reference to Liu et al. [15]). After injection, 20 µL of penicillin/streptomyces should be added to prevent bacterial contamination and then sealed with medical grade breathable tape, after which the eggs should be returned to the incubator. When the recipient chicken embryos develop to 7.5/12.5/18.5 d (HH 32/HH38–39/HH44–45), the embryos are separated with fine forceps under a stereomicroscope and the migration of GFP-PGC is observed using a stereoscopic fluorescence microscope.

### 2.11. Statistical Analysis

Data are presented as the mean ± SEM. Statistical analyses were performed using SPSS (version 25.0, Chicago, IL, USA) and GraphPad Prism (version 8; GraphPad Software, San Diego, CA, USA), histograms were drawn, and statistical significance was set at *p* < 0.05.

## 3. Results

### 3.1. Phenotypic Characteristics of the Combination of Activators and Inhibitors in an Insulin-Based Culture Medium

**Figure 1:**
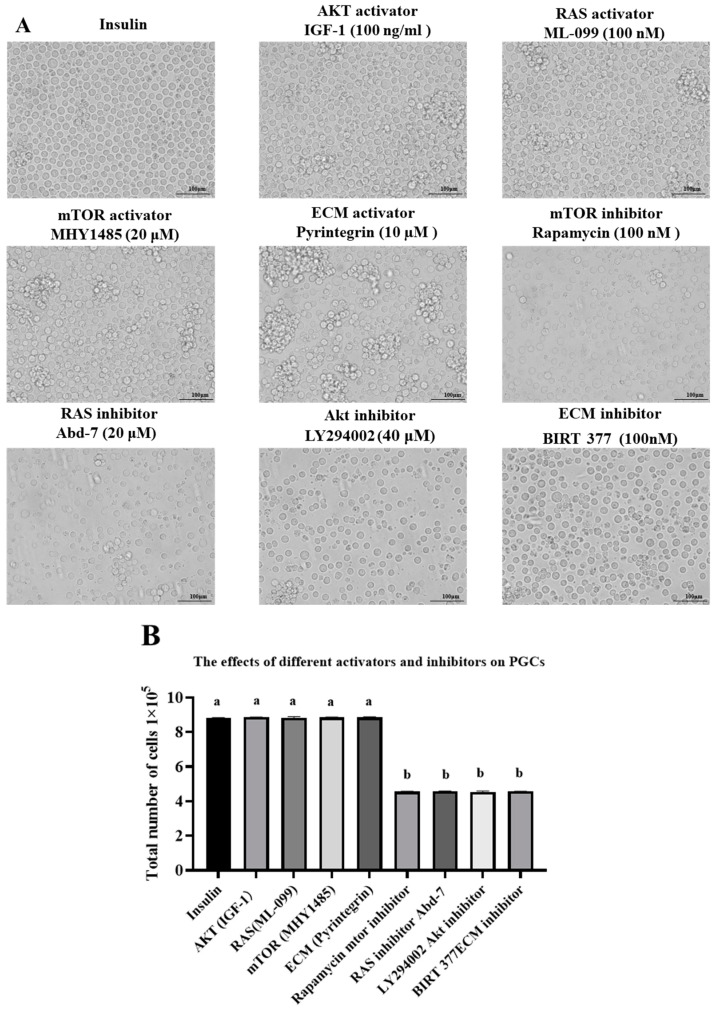
(**A**) Effects of different activators and inhibitors on the morphology of PGCs; scale bar = 100 μm. (**B**) Cell count statistics results. Data are expressed as the mean ± SD of 3 independent experiments. Values within a column followed by different superscript letters differ significantly (*p* < 0.05).

Building upon the existing research findings of our laboratory, it has been established that insulin deficiency leads to a downregulation in the AKT, ECM (extracellular matrix–cell interactions), mTOR (mechanistic target of rapamycin), and RAS (rat sarcoma) signaling pathways. To further investigate the function and regulatory mechanisms of these pathways in related biological processes, PGCs were cultured with specific activators and inhibitors targeting these four signaling pathways, and their survival and morphological changes were observed. Various activators and inhibitors were added to the insulin-containing culture medium and incubated for 72 h. The morphological changes were observed under the microscope (Figure 1A). The results showed that PGCs and IGF-1 (AKT activator) in the insulin group had a uniform and dense distribution, with regular cell morphology and good growth status. PGCs in the ML-009 (RAS activator), MIHY1485 (mTOR activator), and Pyrintegrin (ECM activator) groups showed a state of cell aggregation. The PGCs in the rapamycin (mTOR inhibitor), Abd-7 (RAS inhibitor), LY294002 (AKT inhibitor), and BIRT 377 (ECM inhibitor) treatment groups were relatively sparse, and the cell morphology was severely damaged, with most cells losing their normal morphology. Statistical analysis of cell numbers was performed on PGCs from the different treatment groups (Figure 1B), and the results showed that the cell survival rate of the insulin group was not significantly different from that of the activator group but was significantly higher than that of the other inhibitor groups. The results of microscopic observation and statistical analysis of cell counts indicate that activators have no significant promoting effect on the survival and growth of PGCs, while inhibitor treatment has a negative effect on the survival and morphology of PGCs, leading to changes in cell morphology and a decrease in survival rate.

### 3.2. Effect on PGC Morphology and Proliferation of the Addition of Different Activators Without Insulin

**Figure 2:**
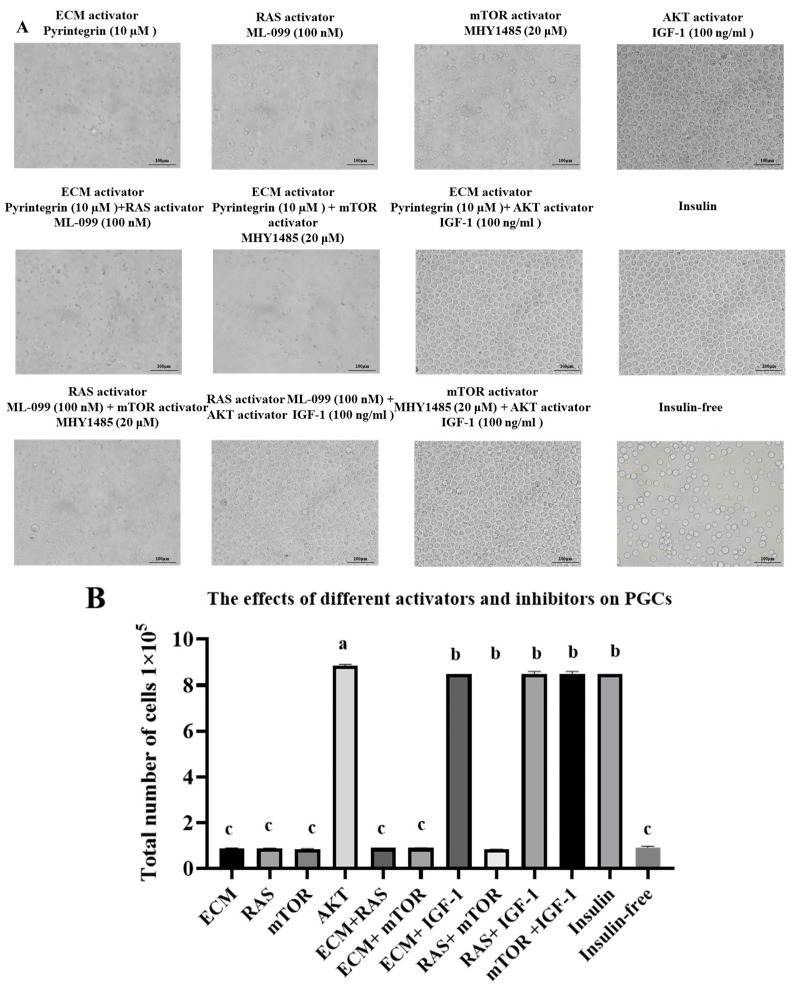
(**A**) Effects of different activators on the morphology of PGCs; scale bar = 100 μm. (**B**) Cell count statistics results. Data are expressed as the mean ± SD of 3 independent experiments. Values within a column followed by different superscript letters differ significantly (*p* < 0.05).

To investigate the effects of different signaling pathway activators and their combined effects on the morphology and survival of PGCs, different activators were added to the culture medium without insulin and cultured for 72 h. The PGCs were observed under the microscope and the results were as follows (Figure 2A): The PGCs in the ECM + RAS, ECM + mTOR, and RAS + mTOR groups treated in combination showed decreased cell density, disturbed cell morphology, and shrinkage. PGCs treated with IGF-1, insulin, ECM + IGF-1, RAS + IGF-1, and mTOR + IGF-1 showed high cell density, regular morphology, and significant cell proliferation. Statistical analysis was performed on the cell counts of PGCs in different treatment groups (Figure 2B), and the results showed that there was no significant difference in the insulin and AKT (IGF-1), ECM + IGF-1, RAS + IGF-1, and mTOR + IGF-1 groups, which were significantly higher than the ECM, RAS, mTOR, ECM + RAS, and ECM + mTOR groups.

These results provide important experimental evidence for a deeper understanding of the regulatory mechanisms of the survival and growth of PGCs, as well as clues for further optimization of the culture conditions of PGCs and for studying the interactions between related signaling pathways.

### 3.3. Effect of IGF-1 on PGC Proliferation

**Figure 3:**
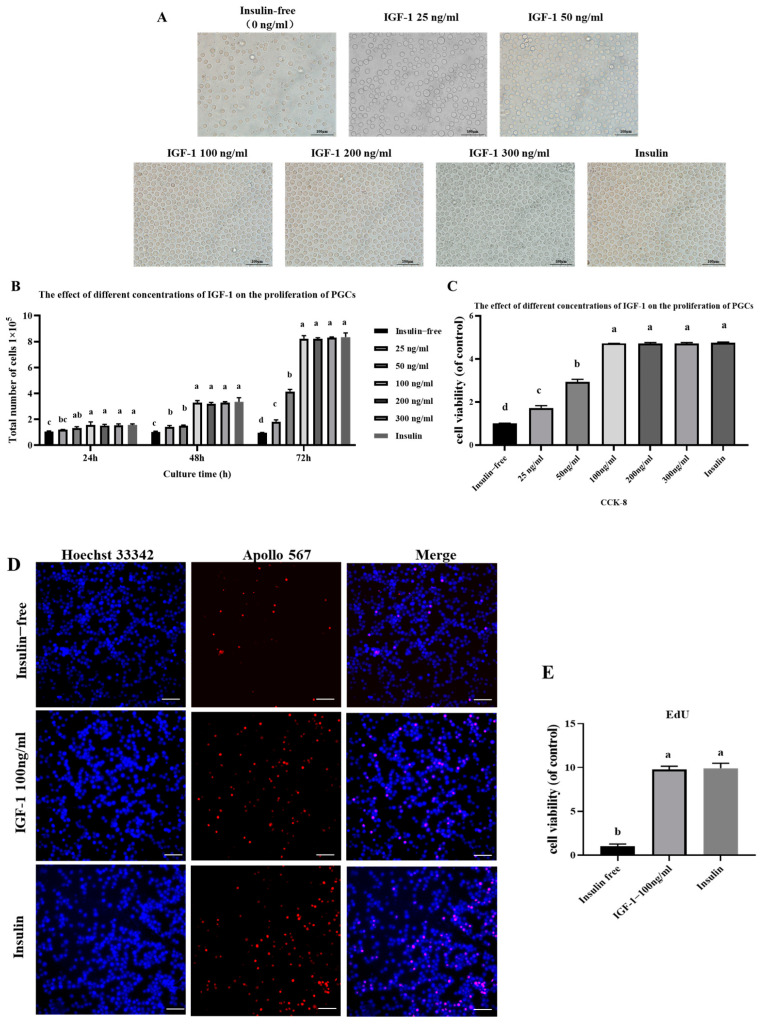
(**A**) Effects of IGF-1 on morphological observation of PGCs; scale bar = 100 μm. (**B**,**C**) Effect of IGF-1 on PGC proliferation. (**D**,**E**) EdU proliferation detection and statistical results. Data are expressed as the mean ± SD of 3 independent experiments. Values within a column followed by different superscript letters differ significantly (*p* < 0.05).

To further investigate the optimal concentration of IGF-1 under insulin-free culture conditions, we added different concentrations of IGF-1 (25 ng/mL, 50 ng/mL, 100 ng/mL, 200 ng/mL, and 300 ng/mL) to the culture medium and cultured for 72 h. Cell morphology was observed under the microscope (Figure 3A). The results showed that the insulin-free group had fewer cells and a sparse distribution; as the concentration of IGF-1 increased, the number of cells showed an increasing trend. The results of cell counting at 24 h, 48 h, and 72 h showed that the insulin-free group and the 25 and 50 ng/mL IGF-1 groups were significantly lower than the other groups at 24 h, 48 h, and 72 h, while there was no significant difference in the total number of cells between the other groups (Figure 3B–E). The above results indicate that 100 ng/mL IGF-1 can replace insulin to promote PGC proliferation.

### 3.4. Effect of IGF-1 on PGC Cell Cycle

**Figure 4:**
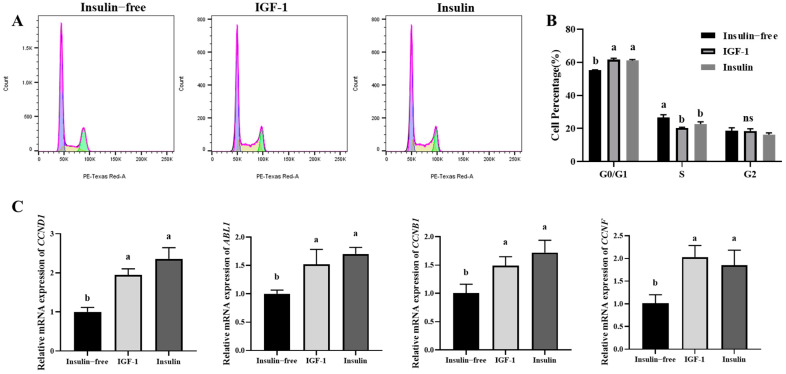
(**A**,**B**) Cell cycle detection and statistical analysis were performed using flow cytometry; (**C**) mRNA expression analysis of cell cycle-related genes, with statistical significance between groups indicated by different letters. Data are expressed as the mean ± SD of 3 independent experiments. Values within a column followed by different superscript letters differ significantly (*p* < 0.05).

To investigate the effects of IGF-1 and insulin on the cell cycle, this experiment first used flow cytometry to determine the percentage of cells in the different treatment groups at each stage of the cell cycle (G0/G1, S, G2). The results showed that the percentage of cells in the insulin-free group was significantly lower than the other two groups in the G0/G1 phase and significantly higher than the other two groups in the S phase, with no difference between the three groups in the G2 phase (Figure 4A,B). The relative mRNA expression levels of the cell cycle genes *CCND1*, *ABL1*, *CCNB1*, and *CCNF* were then determined using qRT-PCR technology. The results showed (Figure 4C) that IGF-1 and insulin had consistent effects on their mRNA expression levels. In conclusion, these results indicate that insulin and IGF-1 treatment can affect the cell cycle progression of PGCs, promote cell entry into S and G2 phases, and upregulate the expression of cell cycle-related genes, suggesting that IGF-1 can replace insulin to promote cell proliferation.

### 3.5. IGF-1 Can Activate the PI3K-AKT Signaling Pathway

**Figure 5:**
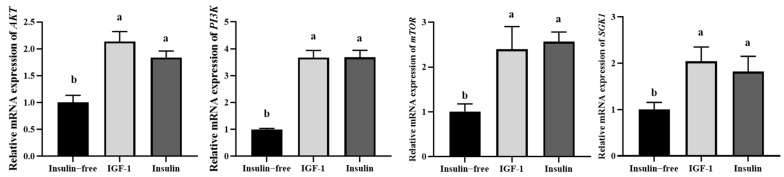
The effect of IGF-1 and insulin media on *PI3K/AKT* genes in PGCs, with statistical significance between groups indicated by different letters. Data are expressed as the mean ± SD of 3 independent experiments. Values within a column followed by different superscript letters differ significantly (*p* < 0.05).

To investigate the effects of IGF-1 and insulin on the expression of PI3K/AKT pathway-related genes in PGCs (Figure 5), qRT-PCR results showed that the relative mRNA expression of PI3K/AKT in the insulin-free group was significantly lower than that in the IGF-1 and insulin groups, while there was no significant difference between the latter two groups. In conclusion, insulin and IGF-1 can affect the expression of molecules related to the PI3K/AKT pathway, and IGF-1 can substitute insulin to promote the activation of this pathway.

### 3.6. IGF-1 Can Inhibit PGC Cell Apoptosis

**Figure 6:**
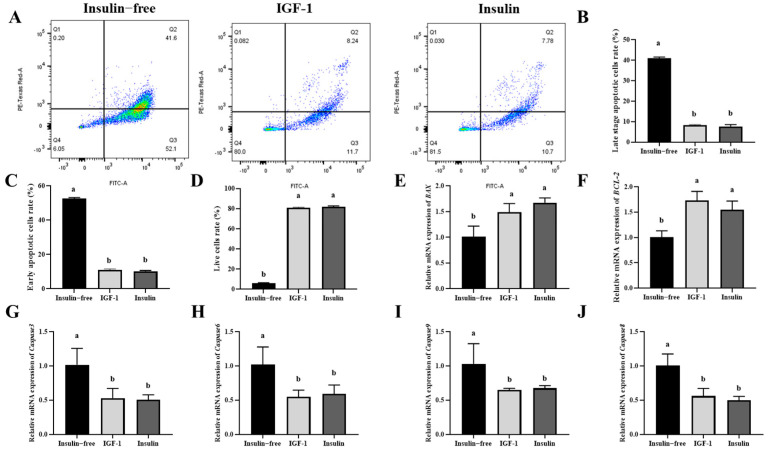
(**A**–**D**) Flow cytometry analysis of apoptosis and statistical analysis of live, early apoptotic, and late apoptotic cell rates. (**E**–**J**) mRNA relative expression analysis of apoptosis-related genes by IGF-1. Different letters in the figure indicate significant differences between groups (*p* < 0.05).

This study investigates the effects of insulin-free controls, IGF-1, and insulin on PGC apoptosis. Flow cytometry was used to assess apoptosis under different treatments. Statistical analysis revealed that cell viability was significantly lower in the absence of insulin compared to IGF-1 and insulin treatment (*p* < 0.05), whereas early and late apoptosis rates were significantly increased (*p* < 0.05). However, no statistically significant differences were observed between IGF-1 and insulin in terms of cell viability and late-stage apoptosis rates (*p* > 0.05) (Figure 6A–D). Furthermore, the relative mRNA expression levels of apoptosis-related genes were detected (Figure 6E–J). The absence of insulin significantly increased the expression of pro-apoptotic genes *Bax*, *Caspase-3*, *Caspase-8*, and *Caspase-9* (*p* < 0.05), while the expression levels of the IGF-1 and insulin groups were similar. Conversely, the expression level of the anti-apoptotic gene *Bcl-2* in the insulin-free group was significantly lower than that in the IGF-1 and insulin groups (*p* < 0.05), and no significant difference was observed between the latter two groups.

In summary, the findings indicate that, in comparison with the insulin-free controls, both IGF-1 and insulin have the capacity to inhibit PGC apoptosis, with the effects of both IGF-1 and insulin being consistent.

### 3.7. IGF-1 and Insulin Can Inhibit the Initiation and Progression of Ferroptosis in PGCs

**Figure 7:**
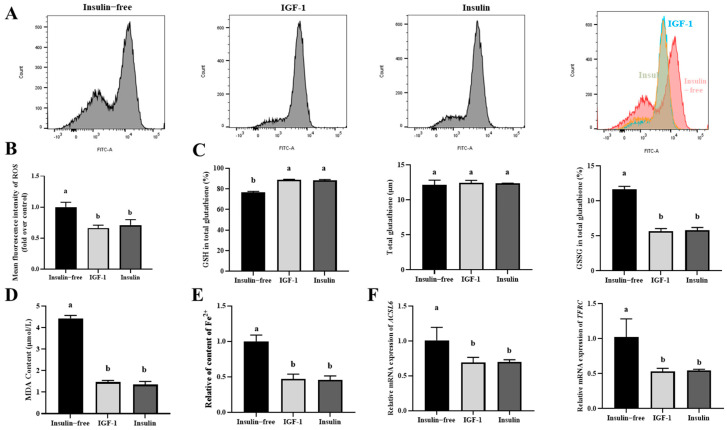
(**A**,**B**) Flow cytometric analysis of the effect of IGF-1 on ROS and fluorescence intensity statistics; (**C**) statistical analysis of GSH/GSSG content; (**D**,**E**) the effect of IGF-1 on MDA and Fe^2+^ content; (**F**) the effect and relative expression analysis of IGF-1 on the mRNA expression of ferroptosis-related genes *ACSL6* and *TFRC*. The significant differences between groups are indicated by different letters in the figure (*p* < 0.05).

To investigate the effects of insulin-free controls, IGF-1, and insulin on ferroptosis in PGCs, intracellular ROS levels were first measured. The results showed that the average fluorescence intensity of ROS was significantly higher in the insulin-free controls compared to the IGF-1 and insulin (*p* < 0.05), with no significant difference between the latter two (Figure 7A,B). These findings suggest that the insulin-free condition leads to elevated ROS accumulation in PGCs, whereas IGF-1 and insulin treatment effectively mitigate ROS levels. Next, the intracellular content of GSH and GSSG were determined, and the results showed no significant difference in total glutathione; the GSH levels in the insulin-free treatment were significantly lower than those in the IGF-1 and insulin treatments (*p* < 0.05); the GSSG of the insulin-free treatment was significantly higher than that of the IGF-1 and insulin treatment (*p* < 0.05), while there was no significant difference between the IGF-1 and insulin (Figure 7C). This suggests that insulin-free conditions may lead to a decrease in intracellular GSH levels and an increase in GSSG levels. IGF-1 and insulin treatment help to maintain the balance of intracellular GSH and GSSG. The MDA and Fe^2+^ contents under insulin-free conditions were significantly higher than those of IGF-1 and insulin conditions (*p* < 0.05) (Figure 7D,E), while there was no significant difference between IGF-1 and insulin. Further detection of the relative mRNA expression levels of ferroptosis genes *ACSL6* and *TFRC* showed that they were significantly lower in the insulin-free condition compared to IGF-1 and insulin conditions (*p* < 0.05) (Figure 7F), while there was no significant difference between IGF-1 and insulin.

In conclusion, insulin-free conditions can lead to an increase in ferroptosis levels in PGCs, as manifested by an increase in ROS, MDA, and Fe^2+^ contents, an imbalance in GSH/GSSG balance, and a decrease in ferroptosis-related gene expression; IGF-1 and insulin treatment can alleviate iron death and have a protective effect on PGCs, suggesting that IGF-1 can replace insulin.

### 3.8. Effects of IGF-1 and Insulin on Key Gene Expression and Migration in Chicken PGCs

**Figure 8:**
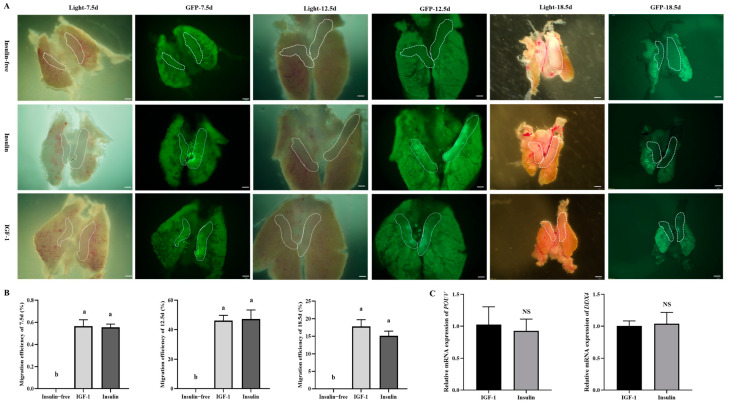
Effects of IGF-1 and insulin treatment on the expression and migration ability of key genes in chicken PGCs. (**A**) Migration images of GFP-PGCs from insulin-free controls, IGF-1, and insulin to the gonads of recipient chicken embryos; (**B**) PGC migration rate in insulin-free controls, IGF-1, and insulin; (**C**) the effects of IGF-1 and insulin on pluripotency and marker genes. The significant differences between groups are indicated by different letters in the figure (*p* < 0.05), NS: not significant.

The two key features of chicken PGCs are the simultaneous expression of pluripotency and germ cell marker genes and the ability to migrate through the bloodstream to the gonads, where they become established [16]. To investigate whether insulin-free, IGF-1, and insulin treatments affect the migratory ability of chicken PGCs, this study cultured GFP PGCs in three different media and injected them into the blood vessels of 2.5-day-old chicken embryos. After hatching at 7.5/12.5/18.5 days, the gonads of the recipient embryos were dissected and the migration of the PGCs was observed under the microscope. The results showed that both IGF-1 and insulin successfully stimulate the migration of PGCs to the recipient gonads, with no significant difference in migration efficiency (Figure 8A,B). Still, the insulin-free group failed to migrate successfully. In addition, further detection of the expression of pluripotency and germ cell-related genes in IGF-1 and insulin showed no significant difference in the expression levels of *POUV* and *DDX4* genes between the two groups through qRT-PCR analysis (Figure 8C). These results indicate that IGF-1 and insulin have no significant effect on the expression and migration ability of key genes in chicken PGCs, suggesting that they play similar roles in the development of PGCs.

### 3.9. Effects of IGF-1 and Insulin on the Efficiency of Establishing PGCs Cell Lines

**Figure 9:**
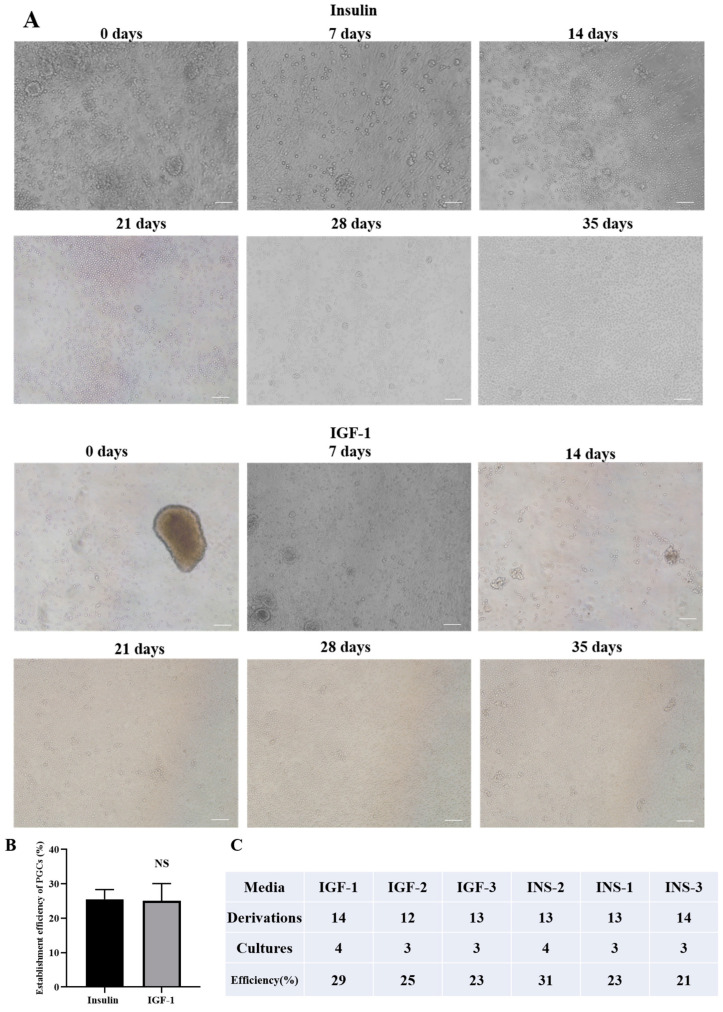
Effects of IGF-1 and insulin on the efficiency of establishing PGCs cell lines. (**A**) Representative images at different time points during the establishment of chicken PGCs showing the changes in cell proliferation; (**B**,**C**) statistical results of IGF-1 and insulin system establishment efficiency. NS: not significant.

Research has shown that bone morphogenetic protein 4 (BMP4) can substitute Activin A under non-clonal conditions, but there are differences in their effects under clonal growth conditions [5]. To investigate the effects of IGF-1 and insulin on the establishment efficiency of PGCs, in this study isolated primary PGCs from the gonads and cultured them. The primary cells with fewer miscellaneous cells could be established about 35 days after isolation (Figure 9A), and the final time to establish all cell lines was 77 days. The number of PGCs exceeded 1 × 10^6^, indicating the establishment of the PGC system. Microscopic observation showed that the density of PGCs gradually increased with increasing culture time. There was no statistically significant difference between the two groups (*p* > 0.05). The above results indicate that in the culture system of PGCs, IGF-1 and insulin have similar effects in promoting cell growth and establishment. IGF-1 can replace insulin to optimize the culture and establishment of PGCs.

## 4. Discussion

This study investigates the effects and potential mechanisms of small-molecule compounds on chicken PGCs based on previous transcriptome data. The research results indicate that under feeder-free cell culture conditions, FGF2, IGF-1, and Activin A are sufficient to promote the derivation, expansion, and clonal growth of chicken PGCs. IGF-1 can significantly promote the proliferation of chicken PGCs and reduce intracellular iron death and apoptosis. In addition, the two culture systems of IGF-1 and insulin have consistent effects on the expression, migration ability, and establishment efficiency of PGC key genes, indicating that IGF-1 can replace insulin.

Research has shown that insulin affects lipid metabolism in goose liver cells through the PI3K/AKT signaling pathway [17], and inhibition of the PI3K signaling pathway can lead to reduced insulin secretion in adipocytes, indicating the importance of PI3K in mediating the insulin response [18]; insulin can also regulate the integrin receptors of the ECM [19]. The ECM signaling pathway can regulate various biological functions such as cell survival, proliferation, adhesion, and migration [20,21,22]. Insulin can also activate the Ras signaling pathway [23,24]. Once activated, Ras acts as a molecular switch, progressively activating Raf and the MAPKs MEK, ERK1, and ERK2 to convert upstream tyrosine phosphorylation into a serine kinase cascade response [25,26]. Insulin can also control protein synthesis through the protein kinase mTOR. mTOR is a member of the PI3K family [27,28,29]. Our research results indicate that other signaling pathway activators cannot promote the proliferation of PGCs, and only IGF-1 can replace the function of insulin and promote proliferation.

IGF-1 can promote the proliferation of human uterine leiomyoma cells through the PI3K/AKT/mTOR pathway [30]. In addition, IGF-1 can regulate the proliferation of muscle, epithelial cells, dental pulp cells, chondrocytes, and neurons [31,32,33,34,35]. These studies collectively suggest that IGF-1 plays a critical role in promoting cell proliferation in various cell types. The effect of IGF-1 on proliferation is mainly achieved by altering the proliferation cycle. Research has shown that IGF-1 can accelerate cell cycle progression, increase DNA synthesis, and promote angiogenesis in cell culture experiments [36]. IGF-1 can promote myoblast proliferation by shortening the G1 and S phases of cellular DNA [37]. Research has shown that IGF-1 can regulate the re-entry of ventricular myocytes into the cell cycle and induce cell cycle arrest and inhibition of cell proliferation in DLBCL cells [38,39]. Insulin stimulates the proliferation of neural stem cells [40] and can also promote osteoclast proliferation by increasing the cell cycle and inhibiting apoptosis [41]. IGF-1 and insulin play a consistent role in promoting cell proliferation and increasing the cell cycle.

Research has shown that IGF-1 can prevent oxidative stress [42]. IGF-1 can sense changes in reactive oxygen species (ROS) levels and regulate the insulin signaling pathway [43]. It protects the heart muscle by reducing ROS damage to cells [44], while also effectively preventing oxidative stress in neurons and intestinal epithelial cells [45,46,47]. IGF-1 can significantly reduce the activity of CAT, SOD, GSH, and GSH px in vitro and in vivo [48]. In this study, we found that the levels of ROS and GSSG decreased in chicken PGCs treated with IGF-1 and insulin, indicating that both treatments can effectively alleviate oxidative stress. In addition, iron poisoning is a regulatory cell death caused by iron-dependent lipid peroxidation, which ultimately leads to cell death [49]. We also found that after treatment with IGF-1 and insulin, the levels of lipid peroxides and Fe^2+^ were significantly reduced, further indicating effective inhibition of oxidative stress and ferroptosis. IGF1 can promote the proliferation of hematopoietic stem cells and mitochondrial oxidative metabolism and also inhibit ferroptosis [50]. Our research results are consistent with previous studies.

Reactive oxygen species are closely related to cell apoptosis [51]. ROS induces cell apoptosis by activating Caspase-3 [52]. In addition, studies have shown that IGF-1 can reduce apoptosis of myocardial cells and rat muscle cells [53,54]. IGF-1 inhibits apoptosis through the PI3K/AKT signaling pathway [55,56,57]. Members of the Bcl-2 protein family participate in various cellular activities by forming dimers and regulating cell apoptosis. Bax is a pro apoptotic member of the Bcl-2 family. After the interaction between Bax and Bcl-2, mitochondrial membrane permeability changes, transmembrane potential is lost, and cytochrome C and other proteins are released. The release of cytochrome C recruits the precursor of Caspase-9 in the cytoplasm, promoting its binding to form apoptotic bodies. Activated Caspase-9 can activate other Caspases such as Caspase-3 and Caspase-7, thereby inducing cell apoptosis [58]. Our research results indicate that IGF-1 and insulin can inhibit the apoptosis of chicken PGCs, and their effects are consistent.

As key cells carrying genetic material, PGCs not only co-express pluripotency markers and early germ cell marker genes, but also possess the biological properties of directed migration and colonization in the gonads [5]. In this study, GFP-PGCs cultured with IGF-1 and insulin were able to migrate to gonadal tissue even after transplantation into HH13–16 recipient chicken embryos. There was no statistical difference in the ability of the two groups of PGCs to migrate to the gonads, indicating that there was no significant difference in the effect of the two culture systems on the ability of PGCs to migrate. By detecting gene expression levels, there was no significant difference in PGCs between the two culture systems. Research has shown that BMP4 can replace Activin A under non-cloning conditions, but there is a difference in line establishment efficiency compared to Activin A under cloning conditions [5]. Therefore, we performed primary cell line culture, and our results showed that the efficiency of IGF-1 and insulin culture systems was consistent, indicating that IGF-1 can replace insulin in the process of PGCs line establishment.

## 5. Conclusions

This study investigated the effect of IGF-1, which replaces insulin, on chicken PGC culture. Under feeder-free culture conditions, FGF2, IGF-1, and Activin A can effectively promote the proliferation of PGCs and significantly inhibit ferroptosis, cell apoptosis, and oxidative stress. IGF-1 shows consistent biological effects with insulin in marker gene expression, migration ability, and cell line establishment, indicating that IGF-1 can replace insulin in PGCs culture systems. Optimization of the IGF-1-mediated culture system helps improve system stability, thereby enhancing the controllability and reproducibility of PGCs in vitro culture.

## Data Availability

Data presented in this study are available upon request from the corresponding author.

## Supplementary Materials

The following supporting information can be downloaded at: https://www.mdpi.com/article/10.3390/genes16050481/s1, Table S1: Real-time PCR primer sequences.
