# Supplementary material for: Functional Equivalence of Insulin and IGF-1 in the In Vitro Culture of Chicken Primordial Germ Cells
Source: Genes (Basel). 2025 Apr 24;16(5):481. doi: 10.3390/genes16050481 (PMC12110881; doi:10.3390/genes16050481)
Supplement: Supplementary file 1 [file genes-16-00481-s001.zip › genes-3579830-SI.pdf]

Table S1. Real-time PCR primer sequences.

Function Classification	Target Gene	Primer Sequence (5′ → 3′)	Length
Pluripotent genes	*POUV*	F: GTTGTCCGGGTCTGGTTCT<br>R: GTGGAAAGGTGGCATGTAGAC	189bp
PGCs marker genes	*DDX4*	F: CAGACCGCATGCTTGATATG<br>R: CAGCCAGCCTCTGAACTTCT	135bp
	BCL-2	F: CCAAGCAAAAAGAGGAGTCACG<br>R: ACCGTTATACCTAATGCAGCCA	120bp
	Caspase-3	F: CTGAAGGCTCCTGGTTTA<br>R: TGCCACTCTGCGATTTAC	104bp
	Caspase-6	F: CCTACACCAACCACCAC<br>R: TCTGCCAAAGTCCCAC	188bp
	Caspase-8	F: AAGGAAGCGGGAAGAT<br>R: GATACCTGAACGGAGACAC	215bp
	Caspase-9	F: ATTCCTTTCCAGGCTCCATC<br>R: CACTCACCTTGTCCCTCCAG	130bp
	BAX	F: TCCATTCAGGTTCTCTTGACC<br>R: GCCAAACATCCAAACACAGA	119bp
PI3K-AKT pathway	*PI3K*	F: CTTCTGGAGTCCTATTGTCG<br>R: CACCTTCTGGGTCTCATCTT	132bp
	AKT	F: GCCGTGAGCCCAGTTAGG<br>R: AGCTACTTATGGCTGCGGGA	153bp
	mTOR	F: AACCACTGCTCGCCACAATGC<br>R: CATAGGATCGCCACACGGATTAGC	120bp
	SGK1	F: ACAAGCAGCCCTATGACA<br>R: TCTCGCAGAGTTGGTAAT	167bp
Cell cycle	*CCND1*	F: TTTGTTCGGCTCGAAGAGAGC<br>R: TCATCGCCAAGGGGAAAACT	124bp
	ABL1	F: AGCTGCCGCTGCTCC<br>R: TTAGCGAAGGCCAAAGCAAC	110bp
	CCNB1	F: CTGCTTTCCGTACCAATGGC<br>R: CAGTCCGTTTTCTTGGGCAC	176bp
	CCNF	F: TTCAAGCCTCCCGTCCTAT<br>R: ACTTCCACCAGCCAGTCAAC	100bp
Ferroptosis	*ACSL6*	CTGTTGGCTCCTCCTACTGC<br>CAGCTCCATGAGAACTGCCA	145bp
	TFRC	F: TGGAGACTCCTGATGCTATCGT<br>R: GTGAAGCCACGACCTTCTGT	120bp
Internal reference	*β-actin*	F: CAGCCATCTTTCTTGGGTAT<br>R: CTGTGATCTCCTTCTGCATCC	169bp
